# Can Tobacco Control Be Transformative? Reducing Gender Inequity and Tobacco Use among Vulnerable Populations 

**DOI:** 10.3390/ijerph110100792

**Published:** 2014-01-07

**Authors:** Lorraine Greaves

**Affiliations:** 1 British Columbia Centre of Excellence for Women’s Health, E311 4500 Oak Street, Box 48, Vancouver, BC V6H 3N1, Canada; E-Mail: lgreaves@cw.bc.ca; Tel.: +1-604-875-2633; Fax: +1-604-875-3716; 2 Faculty of Medicine, School of Population and Public Health, University of British Columbia, Vancouver, BC V6T 1Z4, Canada

**Keywords:** gender, gender-transformative, tobacco use, tobacco exposure, women, men

## Abstract

Tobacco use and exposure is unequally distributed across populations and countries and among women and men. These trends and patterns reflect and cause gender and economic inequities along with negative health impacts. Despite a commitment to gender analysis in the preamble to Framework Convention on Tobacco Control there is much yet to be done to fully understand how gender operates in tobacco control. Policies, program and research in tobacco control need to not only integrate gender, but rather operationalize gender with the goal of transforming gender and social inequities in the course of tobacco control initiatives. Gender transformative tobacco control goes beyond gender sensitive efforts and challenges policy and program developers to apply gender theory in designing their initiatives, with the goal of changing negative gender and social norms and improving social, economic, health and social indicators along with tobacco reduction. This paper outlines what is needed to progress tobacco control in enhancing the status of gendered and vulnerable groups, with a view to reducing gender and social inequities due to tobacco use and exposure.

## 1. Introduction

Gendered disparities and inequities in tobacco use are an issue in high income countries as well as low and middle income countries, as they highlight the unequal distribution of smoking (and exposure to smoking and smoke) among and between men and women, boys and girls, and the health consequences of these patterns. However, the picture is complex, with more than one variable affecting these patterns, making tobacco control responses to such disparities and inequities complicated to design and difficult to mount [1]. In high income countries, factors linked to smoking are low education and low socioeconomic status (SES), mental health issues, being lesbian, gay, bisexual or transgendered, or being members of sub-populations such as Aboriginal or indigenous persons, or particular ethnic groups. Globally, as the tobacco industry efforts and the consequent epidemic shifts to the large populations in low income countries, the picture of inequity caused by tobacco use becomes more stark, with over nine million people expected to die annually by 2020 due to tobacco use, with 80% or over seven million of them in developing countries [2]. Cross-cutting all of these determinants and contributing factors to tobacco use and exposure is gender, a key dimension for analyzing both use and exposure to tobacco, and for assessing the impact of policy, programs and other initiatives [3,4,5,6,7,8,9,10,11,12,13]. 

While males are typically the first users of tobacco in a culture, they are often followed by females [14]. Indeed, the use of tobacco among girls and women is quickly rising across the world, while male use has peaked and is in decline [15]. Unfortunately, acknowledgement of gender and the impact of these gendered trends are often missing in assessments of disparity, inequality and policy impact, and in research design, treatment and prevention in tobacco control [16]. This is despite the obvious importance of both gender and sex as factors in explaining and assessing the impact of tobacco and some clear directives in international documents recommending this approach. For example the Preamble to the WHO-Framework Convention on Tobacco Control, the world’s first international public health treaty, identifies that WHO is: “Alarmed by the increase in smoking and other forms of tobacco consumption by women and young girls worldwide and keeping in mind the need for full participation of women at all levels of policy-making and implementation and the need for gender-specific tobacco control strategies” [17]. Previously, the 1999 Kobe Declaration from the international conference on Tobacco and Health in Kobe, Japan identified the need to halt the tobacco epidemic among women and youth [18]. In addition, there are clear international directives on the importance of considering gender in overall health and development of global societies [19].

## 2. Gender and Tobacco Control: Key Documents

This article reviews the record on gender and tobacco control, examines the concept of gender in more detail based on gender theory, literatures and reports and recommends some actions to improve research, policy and program initiatives in tobacco control. This article aims to raise the bar by suggesting that we engage with gender not only by inserting it acritically into tobacco control, but by engaging more deeply with its various meanings and impacts and focusing tobacco control initiatives on remedying some of the broader gender and social inequities linked to tobacco use. This is posed as a challenge for the next decade of tobacco control, and if taken up, could have a major impact not only the health of women and men worldwide, but also their social and economic status as well. 

Gender has been widely studied at the World Health Organization (WHO) with a view to understanding how gender plays out in affecting health and equity, especially among girls and women. The WHO Gender Policy was established to guide agencies, governments and programs across the world in naming, understanding, and responding to gender issues. The recent WHO Commission on the Social Determinants of Health not only reiterated gender as a key social determinant of health, but delved into the impact of gender more thoroughly, via its Women and Gender Equity Knowledge Network, which submitted a final report to the WHO Commission in 2007 [20]. This report states that “taking action to improve gender equity in health and to address women’s rights to health is one of the most direct and potent ways to reduce health inequities and ensure effective use of health resources” [20].

At the same time, high income countries such as Canada have had gender analysis policies for some time [21], intended to make sure that the impacts of all policy be assessed for their gendered impacts. Unfortunately, a recent audit of the use of this policy indicated it has been a failure [22]. In the USA, a related 1993 edict (the NIH Revitalization Act) to include gender in all National Institutes of Health (NIH) funded research met with mixed results, resulting in compliance with the Act but little analysis of the resulting data [23]. These examples indicate that while a need has long been identified by national and global bodies, and various approaches have been tried, it has been remarkably difficult to meaningfully integrate gender and gender analyses into the development of health policy. 

Why this is so is a scientific mystery. Certainly there has been a long tradition of ignoring gender in both research, education and policy, only relatively recently being addressed in research funding and training, by decision making bodies and some journal editors. In addition, there has been a mistaken conflation of “gender” with “women” in many documents and initiatives which may have alienated some from grasping the overarching importance of gender to tobacco control. Finally, despite calls to the contrary, sexism and lack of interest in equality issues continue to dog science and tobacco control similar to many other aspects of human endeavour. However, the obvious relevance of both sex and gender to tobacco control and the growing scholarship on these issues point to the imminent need for its inclusion and study.

Meanwhile, gender theory has progressed, delving into and inviting multiple perspectives on explaining gender disparities, developing better conceptualization, methods and measures of using both sex and gender in research and programming, and reflecting more detailed understandings of gender and its impact on human health [24]. Several guides and books have been issued to assist with integrating gender considerations in health policy [25,26] and research [27,28]. In the field of tobacco, specific gender analyses of Canadian tobacco policy [29], the FCTC Articles [30,31] and global gender and tobacco issues [14,32,33] have been made, along with specific advice on interventions aimed at women and in some cases, men, with a gendered view [34,35,36]. Indeed, the history of gender and tobacco and women and tobacco reporting and research is rich, long and critical [32].

Underpinning these, in addition to the WHO-FCTC Preamble, have been initiatives such as the Kobe Declaration [18], which orders the integration of gender equality into tobacco control, the Convention to Eliminate Discrimination Against Women [37] which mandates a gender perspective into all policies and programs affecting women’s health and which has recently been used by Argentine activists to pressure the government of Argentina on its record on women and tobacco [38] and other advocacy measures imploring tobacco control to take gender more seriously. But actions on these matters have been scant. Warner, in an opening commentary introducing the recent and important special issue of the American Journal of Health Promotion on diverse populations and smoking mentions almost all dimensions of disparity but gender [39]. In the same issue, Perez-Stabel and Benowitz address biological differences in explaining tobacco-related disparities in the USA and do not mention sex, a known element affecting nicotine metabolism and response to therapy [40]. These types of blind spots to both sex and gender and their impact on tobacco use, exposure and control efforts are not unusual, but are highly problematic.

## 3. Conceptual Relevance of Gender to Tobacco Use and Exposure

### 3.1. Gender and Sex

Gender is often conflated with sex and used interchangeably [41], which is often confusing and superficial. Sex refers to the biological aspects of being female or male, and the impact of biology on physiological processes, genetics and body structures [27]. Gender, on the other hand, refers to the myriad cultural and social aspects associated with being female or male in a given context and affects socialization, roles, opportunities, and social and legal regulations. These concepts are often poorly understood and applied in health research, policy and programming, including tobacco control.

It is critical to understand that both gender and sex are continuous variables [27,28], in that humans can have differing levels of sex-related factors such as hormones [42,43] and be influenced in a range of different ways by gendered expectations in any given society that often reflect social, cultural, religious and legal systems. Examples of sex related factors in tobacco use include sex-specific responses to nicotine and rates of absorption of nicotine and nicotine replacement drugs [44] and even their potential impact on standard tests for dependency on smoking or nicotine [45]. While sex is, when acknowledged in research, most often demarcated as “male” or “female” as two discreet categories, this practice does not reflect the mash of elements such as different levels of hormones, amounts of muscle mass, sizes of body parts or numerous chromosomal combinations other than XX or XY. 

Nor is gender dichotomous, with humans fitting neatly into two categories of “men” and “women”. The array of gendered bodies and lives also include intersex, “third sex”, transsexual and transgendered individuals, along with gay, lesbian, bisexual and ‘two-spirited’ people. These varied identities are expressed and recognized in culturally different ways, affected by politics, time, place, social norms and values. Hence, it is useful to delineate gender into several elements in order to understand how it functions in context, affecting health and in affecting exposure to and use of tobacco. Examples of gender related factors affecting tobacco use, exposure and responses to tobacco control include gendered advertising and messaging, gender-specific tobacco product development, differential responses to health promotion, and inequitable power in couples and households, poverty levels and experiences of violence and trauma [6,34,46,47,48]. Together, sex and gender represent a powerful set of influences that often interact, and assist greatly in explaining and responding to tobacco use and exposure among both females and males, and all genders. Without fully engaging and understanding these factors, tobacco control, including policy, practice and regulation, will fall short of effectively shifting prevention, cessation and initiation trends.

#### 3.1.1. Gender Identity

Gender identity refers to the manner in which a person regards themselves as male, female or other. Gender identity is formed in response to and in a cultural and temporal context and affects aspirations, behaviours and body image, among other aspects of identity [27]. In relation to tobacco use, identity has been shown to be formed or affected by tobacco use and smoking, both in terms of the rituals and behaviours associated with smoking, as well as absorption of messages and images related to tobacco use [49]. This is likely true for girls, boys, women and men. In some cases, culturally imposed ideas about femaleness or maleness can either inhibit or encourage tobacco use [46,49], as has been illustrated over time by examining trends in introducing tobacco into various cultures. Tobacco companies have repeatedly used the concept of gender identity to promote and develop a range of gender-specific brands for almost 90 years [46,49,50]. 

#### 3.1.2. Gender Relations

Sen and Östlin [20] zero in on gender relations as the pivot on which inequality balances, as gender relations shape everyday experiences of health and access to health care, and reflect power distributions in society. The links between gender, inequity and health are seen as key opportunities or barriers in advancing development. In tobacco use, gender relations are implicated in initiation, maintenance and cessation or reduction patterns, as relational dynamics can be either negative or positive influences in these patterns. For example, tobacco reduction during pregnancy and *post partum* has been shown to be heavily influenced by gender relations; sometime for the better and sometimes not [34,36,48,51]. It is clear that tobacco use and reduction occurs in context, and is affected by relationships with partners, [34] family members [52] and friends [53] and in specific cultural groups [54].

#### 3.1.3. Institutional Gender

Institutional gender reflects the rules and laws that affect the distribution of power ascribed to genders in social institutions such as education, politics, media, religion or family. These institutions shape the norms that define opportunities and expectations of individuals by gender [27]. The limits or opportunities affect social practices such as labour force roles and participation, gender roles at home, in media and public life, level of income and freedom of movement. In tobacco use, these influences affect tolerance for use among various subpopulations [55] abilities to purchase tobacco [56] or the acceptability of using tobacco in public [46,49] or the overall vulnerability to tobacco use among subpopulations [57].

#### 3.1.4. Options for Action on Gender

This brief overview of the concepts of sex and (the various aspects of) gender indicates that there is rich opportunity for assessing, designing or improving tobacco control initiatives against this backdrop. However, as in any other health, economic or social issue, there are options for addressing gender that range from gender blind (ignoring gender altogether) to gender transformative (changing negative underlying gender norms along with tobacco control). In between are possibilities for gender-sensitive or gender-specific approaches such as programs, policies or research that specifically address populations by sex/gender (and/or other diverse characteristics such as socioeconomic status (SES) or indigenous status), but not necessarily in an enlightened and progressive manner. Unfortunately, tobacco control, despite its over 50 year history, remains unlikely to address gender in any form [16]. It is important to note that rectifying this oversight could assist in generating much better results in tobacco control initiatives ranging from prevention to cessation to policy and research. So how can oversights in integrating gender concepts into tobacco control be rectified?

#### 3.1.5. Gender-Transformative Tobacco Control

The call for including gender in tobacco control is fairly long-standing [58,59], as has been the call for integrating gender concerns along with other disparities or inequities [3,5,6,8,9,10,11,60]. Nonetheless, there has been fairly low uptake of gender and/or sex as components of tobacco control initiatives, and almost no engagement with gender theory as it has developed over the past three decades. In some cases gender analysis has been included as an afterthought as opposed to integrated into initial design, or sex/gender exploited to achieve tobacco control goals (such as the common practice of appealing to aspirations of sexual attractiveness to deter girls from smoking, or enlisting women to monitor and police their male partners’ smoking to reduce exposure). However, any simple call for the inclusion of gender has now been overtaken by calls for more complex gender-transformative approaches that would both simultaneously enhance health and status, and also reduce tobacco use. While as yet largely untested, this approach is more likely to address overarching health inequities resulting from the confluence of gender, low income or processes of discrimination and other sources of inequity that deeply affect tobacco use, prevalence and exposure. While these issues have been identified as salient for women and girls, given the continuing escalation of global prevalence of tobacco use (in comparison to declining global rates of use for men, globally), and their generally lower social and political status, gender-transformative tobacco control would benefit any gender by addressing negative gender norms.

Greaves and Tungohan specifically called for gender transformative tobacco control in 2007, citing several ways in which such an approach would specifically enhance the progress of the WHO-FCTC and would benefit women in particular [31]. These calls were based on suggestions in the WHO report [61] that delineated “exploiting”, “accommodating” or “transforming” gender as three options in developing health programming. Greaves and Tungohan transferred these concepts to tobacco control using Articles in the WHO-FCTC and illustrated each, in order to generate discussion and initiatives that were ideally transformative, but at the very least accommodating. Exploiting gender in the name of tobacco control was rejected as a regressive and damaging approach. The gender transformative suggestions were more progressive than was normally seen in tobacco control, and unfortunately remain so seven years later. 

Gender transformative tobacco control could include a range of diverse initiatives in all domains of comprehensive tobacco policy environments. For example: including explicit goals of increasing income and household empowerment for women and child tobacco workers by tracking such measures in surveillance and advocating for changes that improve labour options, increase status and lessen vulnerability to abuse: explicitly naming a person’s health and self respect as the prime motivator for prevention or cessation, as opposed to reinforcing gendered norms of sexual attractiveness, masculinity or femininity; focusing on a full range of roles in designing tobacco control initiatives other than reproduction and pregnancy and fetal health (for women) and work (for men) by generating evidence based approaches that include fathering (for men) and work (for women) among the *many* other social roles and experiences that individuals occupy. Finally, avoiding the impulse to “protect” women from tobacco use using rigid cultural or religious norms that limit freedoms as a basis for tobacco control that would be exploitive, not transformative. Examples such as these, and others, need development, experimentation and evaluation, in order to move tobacco control into a more relevant and reflective era. These examples focus on shifting gendered practices by using tobacco control as a tool for so doing. In return, tobacco control will be more relevant, progressive and respected as a prime contributor to reducing health inequity and improving health for all populations. Tobacco control could move past a sole focus on generic tobacco control and/or its often simplistic exploitations of gender in programming. In so doing, tobacco control will be better equipped to face the challenges of an expanding epidemic in low income countries in the coming decades; a gendered epidemic that is complicated by poverty, violence, inequality, structural pressures on economies, vulnerability to globalization and pressing health literacy issues.

## 4. Conclusions

It is clear that tobacco control has largely failed to fundamentally integrate gender into its operation and overlooked the importance of consistent sex and gender analyses over the past few decades in programs, research and policy [16]. But more importantly, mainstream tobacco control has missed decades of scholarship and research on gender that have been shown to have a direct influence on tobacco use and exposure, as well as responses to standard comprehensive tobacco control actions, measures and policies [3,6,7,8,11,45,57,62,63,64,65]. It is a mystery as to how tobacco control continues to escape these influences, especially since the tobacco industry has exhibited a long and rich history of integrating gender into its operations, sales and marketing, resulting in huge successes at transforming social norms [46,49]. In addition, there has been no shortage of global documents exhorting tobacco control to take gender into account, nor a shortage of advocacy and research from the International Network of Women Against Tobacco (INWAT) a 24 year old official non-governmental affiliate of the World Health Organization, dedicated solely to reducing or preventing tobacco use among girls and women. The scholarship on both sex- and gender-related factors affecting tobacco use, exposure, effects, and responses to policy and programming is extensive and powerful [14,32,33,66]. While tobacco control has been largely gender-blind, gender theory has progressed to the point of increasing complexity, reflecting the lived experiences of women and men, boys and girls across the world. Indeed, gender theory has progressed to offer a range of insights for responding to tobacco use, and setting a stage for tobacco control to lead, should its leadership so desire.

Wider efforts to integrate gender theory with health promotion have led to similar calls across a range of other health issues [67]. Gender transformative health promotion is being promoted as a needed improvement on generic “one-size-fits-all” approaches that will address inequities due to gender and other interacting and intersecting factors such as income, power, discrimination and autonomy. As these more sophisticated aims for gender-transformation seep into the health, health promotion and tobacco control agendas, in a context where much of tobacco control is still gender blind, is it possible to jump to this more sophisticated place and raise the bar? Possibly, if *all* relevant science is taken into account, well-established international directives are respected and the Preamble of the WHO-FCTC itself is taken seriously and measured. Perhaps these goals will be reached more quickly if journal editors begin to demand sex and gender reporting in all scientific papers, and offer training to peer reviewers, and existing WHO and national level policies on gender be developed and followed, with evaluations of their impact. Similarly, pressure from funding agencies for applicants to incorporate sex and gender analysis into research proposals or program evaluations is a growing practice, and is a first step in incorporating gender into tobacco control. Assessing and inviting conference presentations at tobacco control conferences to take gender transformation into account would also assist in reaching these goals faster. These are all calls for the tobacco control community to acknowledge a greater responsibility than reducing tobacco use and exposure at any cost, and to endorse and engage with making positive changes in the lives of women and men along the way, as a direct route to reducing the gendered health inequities linked to tobacco. Given the prognosis for the spread of tobacco use across low income countries in the 21st Century and its predicted influence on gendered health and economic inequities, rectifying these oversights is not only urgently required, but also never too late. 
